# Acute ecotoxicology of natural oil and gas condensate to coral reef larvae

**DOI:** 10.1038/srep21153

**Published:** 2016-02-19

**Authors:** Andrew P. Negri, Diane L. Brinkman, Florita Flores, Emmanuelle S. Botté, Ross J. Jones, Nicole S. Webster

**Affiliations:** 1 Australian Institute of Marine Science, Townsville, 4810, Queensland, and Perth, 6009, Western Australia, Australia

## Abstract

Risks posed by oil spills to coral reefs are difficult to evaluate, partially due to the absence of studies that adequately assess toxicity to relevant coral reef species. Here we experimentally tested the acute toxicity of condensate, representing a fraction of light crude oil, to coral (*Acropora tenuis*) and sponge (*Rhopaloeides odorabile*) larvae. The metamorphosis of coral larvae was inhibited at total petroleum aromatic hydrocarbon (TPAH) concentrations of water accommodated fractions (WAF) as low as 103 μg l^−1^, similar to concentrations detected in seawater following large spills. The sensitivity of coral larvae increased by 40% when co-exposed to UV light that they might encounter in shallow reefal systems. Condensate WAF was more toxic to coral larvae than predicted by summing the toxicity of its main components (benzene, toluene, *p*-xylene and napthalene). In contrast, the sensitivity of sponge larvae to condensate WAF (>10,000 μg l^−1^ TPAH) was far less than coral in the presence and absence of UV, but similar to that of other marine invertebrates. While these results highlight the relative sensitivity of coral larvae to oil, further research is needed to better understand and predict the impacts and risks posed by hydrocarbons to tropical reef systems.

Renewed interest in the effects of hydrocarbon spills on sensitive marine and coastal ecosystems was stimulated by two high profile oil spills (‘blowouts’), the Montara well-head platform incident off north west Australia^1^ in 2009, and shortly afterwards, the 2010 Macondo (*Deepwater Horizon)* incident in the Gulf of Mexico^2^,^3^. The Montara incident released ~4,500 m^3^ of medium crude oil^1^,^4^,^5^ into a unique marine biogeographic province, containing biodiversity hotspots such as carbonate reefs, banks and shoals that are spatially related (perhaps causally related) with active and palaeo-hydrocarbon seeps^6^,^7^. The submerged banks and shoals formed by the green alga *Halimeda* spp. and other coralline algae^8^ are particularly abundant in the Timor Sea and their sunlight exposed summit regions are dominated by photoautotrophic scleractinian corals, octocorals, green algae and seagrass^4^, with the deeper (>25–30 m) flanks dominated by filter and particulate feeders including sponges, soft corals and bryozoans^4^. The much larger Deepwater Horizon event released ~780,000 m^3^ of light crude oil, with sub-surface plumes reportedly affecting deep benthic communities, including cold-water coral populations^9^,^10^.

Tropical coral reefs are in decline and additional impacts from human activities, including hydrocarbon pollution, place these ecosystems at further risk^11^. In addition to blowouts, coral reefs can be exposed to hydrocarbons from shipping accidents^12^,^13^ and spills from coastal processing facilities. One of the largest documented oil spills into shallow tropical reef environments (the Galeta spill, 1968) involved the release of ~10,000 m^3^ of medium crude oil from refinery storage into the nearshore waters of the Caribbean coast of Panama^14^. This spill had extensive effects on mangroves, seagrasses and corals^14^, with very little evidence of recovery of coral reefs observed five years later^15^.

Among the most vulnerable tropical organisms to oil are the sessile benthic invertebrates which cannot actively avoid exposure. The effects of hydrocarbons on corals is of particular importance as they are the principal habitat-forming species on reefs, and there have been several laboratory- and field-based studies describing the lethal and sub-lethal effects of exposure^12^,^16^,^17^,^18^. For other important taxa such as marine sponges^19^, which are the primary filter-feeding taxa on reefs and act as a critical link between benthic and pelagic habitats, there have been fewer studies. Also, very little is known about the effects of hydrocarbons on early life history stages of reef invertebrates. These stages are particularly important as successful recruitment underpins recovery and resilience of reefs following disturbance^20^,^21^. Pioneering field studies in the Red Sea described significant decreases in the reproductive output of corals and settlement and metamorphosis of pelagic coral larvae at a site chronically contaminated with crude oil^22^,^23^. These early observations have been complemented by ~12 laboratory studies investigating effects of hydrocarbons (gasoline, condensate, fuel oil, crude oil and lubricants) on various life stages of coral (including early release of larvae, fertilization, embryo development, larval survival and metamorphosis and juvenile growth, summarised in Table 1). Some of the laboratory studies indicated early life stages of coral may be sensitive to petroleum hydrocarbons, but effect threshold concentrations ranged by over three orders of magnitude between studies, species and life stages. The only equivalent study on sponges was with the encrusting sponge, *Crambe crambe*, and indicated larvae were possibly more sensitive to hydrocarbons than most coral larvae (Table 1).

In order to predict risk to marine species from oil spills and blowouts it is necessary to understand both the potential exposure and likely effects amongst a range of different organisms. The wide range of sensitivities of early life stages of corals and sponges to hydrocarbons in Table 1 could be due to differences in the type of oil and the experimental conditions, including exposure duration. The variance could also be due to differences in the life history stages used and also to intrinsic differences in the sensitivity of different species or taxa. This latter source of variation is particularly important for the derivation of environmental quality criteria and for risk assessment purposes, as it is the basis of species sensitivity distribution (SSD) analyses (i.e. probability distribution of some measure of toxicity to a certain chemical in a population)^24^. Comparison of hydrocarbon exposure studies is not always possible, as concentrations are typically expressed in terms of water accommodated fraction concentration (% WAF) or total hydrocarbons (THC), which can vary in composition depending on the hydrocarbon source and the protocol used for WAF preparation^25^. The exposure of coral reef organisms to hydrocarbons following uncontrolled releases has not been reliably reported, but comprehensive water column measurements made during the *Deepwater Horizon* spill provide a suitable reference point for likely concentrations in sub-surface slicks. The spill released a wide range of petroleum hydrocarbons (gas and oil), including n-alkanes, branched alkanes, monoaromatic hydrocarbons (MAHs) and polycyclic aromatic hydrocarbons (PAHs)^26^. Sub-surface total PAHs (ΣPAH_41_) reached 189 μg l^−1^ (~1,300 m deep and <5 km from the platform)^27^, while total benzene, toluene, ethylbenzene and xylenes (collectively referred to as BTEX) reached 78 μg l^−1^ (in samples collected at ~1200 m deep and 6 km from the well head)^26^.

Oil and gas exploration and production in north-west Australia faces a unique conservation challenge, which is to balance the commercial value of the underlying hydrocarbon prospects against the potential for accidental releases during exploration and production and potential loss of conservation value of these unique ecosystems. The toxicity of north-western Australia light crude oil has been examined on several temperate species as well as tropical fish, shrimp and urchin larvae^28^; however, the toxicity to sessile tropical reef invertebrates such as corals and sponges has not yet been described. For tropical ecotoxicology, important environmental variables are water temperature and UV irradiance, which are both higher in low latitude environments. For example, UV irradiance can increase the potency of PAHs through oxygen radical formation and concomitant damage to membranes and DNA^29^. To improve risk assessments, and to better inform decision-making for oil and gas industry and government for oil spill response, we examined the acute toxicity of North West Shelf condensate to coral and sponge larvae. Condensate (also referred to as natural gasoline or distillate) is the hydrocarbon fraction of a gas or light crude well that remains liquid at room temperature and 1 atmosphere. In addition, we examined the effect of simultaneous exposure to UV light, and the toxicity of the four major aromatic components of the water accommodated fraction of condensate to examine their potential contribution to toxicity.

## Results

### Condensate and WAF composition

The chemical characteristics of stabilised condensate were typical of a relatively light petroleum product. Analysis of the GC-amenable hydrocarbons revealed that the condensate contained predominately odd and even *n*-alkanes (most abundant within the *n*-C_5_ to *n*-C_10_ range), branched alkanes, as well as parent and alkyl-substituted cycloalkanes, benzenes and PAHs (Supplementary Fig. S1). The PAHs were characteristically lower in molecular mass distribution compared to heavier oils and primarily included parent and alkyl naphthalenes, fluorene and phenanthrene (Supplementary Table S1). BTEXs and PAHs constituted 2.8% and 0.6% (w/w) of the condensate, respectively.

Solvent extracts of freshly prepared condensate WAF differed considerably in their hydrocarbon profiles compared to the original condensate. No *n*-alkanes or branched alkanes were detected in the WAFs, as assessed by extracting the three major ions characteristic of these compounds (m/z 57, 71, 85) from the GC-MS total ion current chromatogram. Instead, the hydrocarbon composition was dominated by BTEXs, other alkyl-substituted benzenes and the naphthalene series (Supplementary Fig. S2). Some losses of more volatile condensate hydrocarbons due to evaporation likely occurred during the mixing period. The remainder of the condensate formed a visible immiscible slick on the seawater surface after settling. The concentration of total BTEXs accommodated within the seawater fractions for the coral and sponge exposures were 12.7 and 27.8 mg l^−1^, respectively, while total PAH concentrations were 157 and 224 μg l^−1^, respectively (Supplementary Table S2). Total BTEXs and total PAHs in the coral exposure WAF were 55% and 30% lower than those in the sponge exposure WAF, respectively. Such variations may have resulted from greater losses of the volatile and semi-volatile components during preparation and/or sampling of the coral exposure WAF. A summary of individual BTEX and PAH concentrations in condensate and the freshly prepared WAFs used in the exposure experiments are summarised in Table 2. Over the course of the larval exposure experiments, the headspace in the sealed glass incubation vials (~10%) resulted in 35–55% evaporation of TPAH over 24 h and the average of the initial and final TPAH concentrations were subsequently applied as exposures in concentration-response curves.

### Effects of condensate and UV on coral and sponge larvae

**Figure 1:**
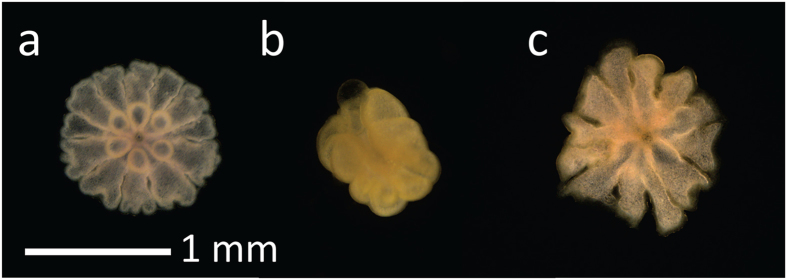
Photomicrographs of 24 h old juvenile corals showing normal post settlement metamorphosis, showing completion of primary and secondary mesentery formation of a single polyp with six tentacles surrounding a mouth, partial and disrupted metamorphosis when exposed to PAHs and WAF condensate where (**a**) control, (**b**), 5,600 μg l^−1^ TPAH in stabilized condensate WAF and (**c**) 34,000 μg l^−1^ benzene WAF.

**Figure 2:**
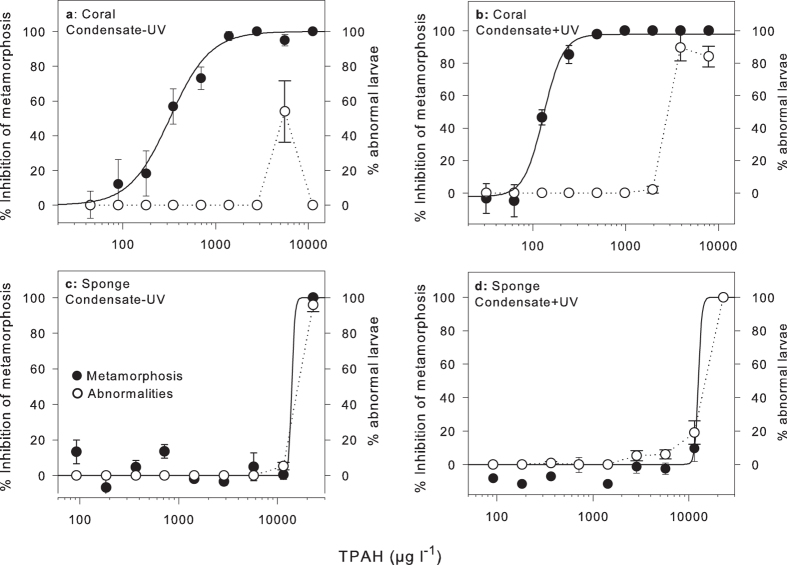
Concentration-response curves for inhibition of metamorphosis of coral and sponge larvae in the presence of condensate WAF (μg l^−1^ TPAH). Closed circles represent mean inhibition (%, relative to control) of coral larval metamorphosis to condensate WAF in the (**a**) absence of UV and (**b**) presence of UV and of sponge larval metamorphosis to condensate WAF in the (**c**) absence of UV and (**d**) presence of UV. Open circles represent mean abnormalities (%) of larvae in the same treatments. Mean ± SE of six replicate exposures. Summary results from these curves can be found in Table 3.

After 24 h in uncontaminated seawater 70–84% of ‘control’ coral larvae underwent successful attachment and metamorphosis in each experiment (Table 3). No difference in metamorphosis success (performance) was observed between control larvae (7–13 days old) over this period (ANOVA F_5_ = 1.4, *p* = 0.24). Coral larvae exposed to condensate WAF over 24 h also exhibited normal settlement and metamorphosis behaviour at low concentrations (<100 μg l^−1^), but this development process became increasingly inhibited at higher condensate concentrations (Fig. 1). Fitting the % inhibition data to a logistic equation allowed calculation of concentrations that inhibited metamorphosis by 10% (IC_10_) = 103 μg l^−1^ and 50% (IC_50_) = 339 μg l^−1^ TPAH (Table 3). Co-exposing coral larvae to high UV for 2 h under otherwise identical conditions inhibited metamorphosis by 50% at a significantly lower concentration of IC_50_ = 132 μg l^−1^ TPAH (p < 0.05, Fig. 2b, Table 3).

Coral larvae that did not metamorphose remained intact up to the maximum concentration tested of 11,100 μg l^−1^ TPAH for 24 h. After the addition of CCA extract to induce metamorphosis and an additional 18 h of development, 54% larvae exposed to ~5,600 μg l^−1^ TPAH exhibited abnormal development (Fig. 2a), characterised by partial metamorphosis in the water column without attachment (Fig. 1b). Similar abnormal development of polyps in the water column was observed for coral larvae exposed to ≥3,900 μg l^−1^ TPAH (Fig. 1b). Several of these abnormal floating polyps were isolated and maintained for an additional 48 h in uncontaminated seawater but did not show any attempts to attach and undergo metamorphosis.

After 24 h in treatment conditions in uncontaminated seawater, 82% and 90% of control sponge larvae were able to successfully attach and undergo metamorphosis (Fig. 2c,d, Table 3). Sponge larvae were far less sensitive to condensate WAF over 24 h than corals, and were not affected by condensate WAF until concentrations exceeded 11,000 μg l^−1^ TPAH (Table 3). Above this concentration larvae appeared either as small spheres or were misshapen, with thin mucoidal strands detaching from the surface. No successful metamorphosis was seen in any of the larvae above 11,000 μg l^−1^ TPAH (Fig. 2c,d) and co-exposure of sponge larvae to UV had no additional effect on metamorphosis.

### Effects of individual MAHs and naphthalene on coral larvae

**Figure 3:**
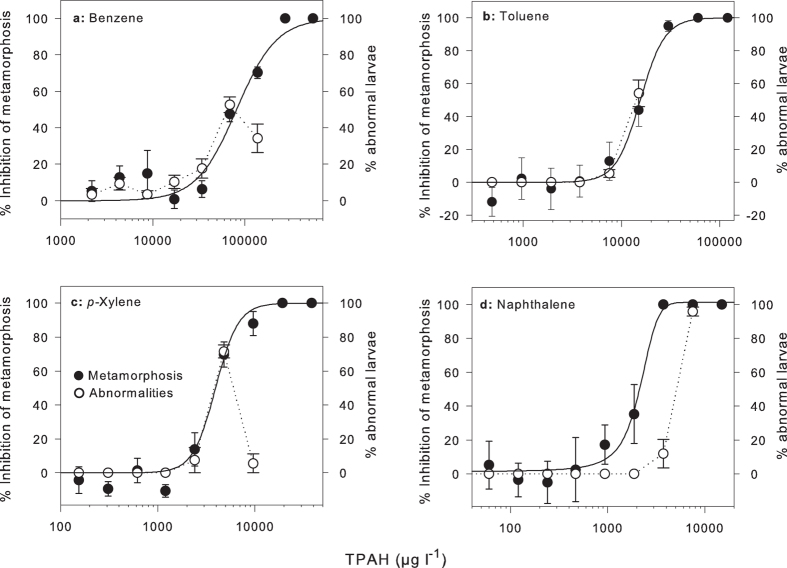
Concentration-response curves for inhibition of metamorphosis of coral larvae in the presence of the major aromatic components found in condensate WAF (μg l^−1^). Closed circles represent mean inhibition (%, relative to control) of coral larval metamorphosis to (**a**) benzene, (**b**) toluene, (**c**) *p*-xylene and (**d**) naphthalene. Open circles represent mean abnormalities (%) of larvae in the same treatments. Mean ± SE of six replicate exposures. Summary results from these curves can be found in Table 3.

Four major aromatic components of condensate WAF, benzene, toluene, *p*-xylene and naphthalene, all individually inhibited settlement and metamorphosis of coral larvae after a 24 h exposure (Fig. 3). Fitting the % inhibition data to a logistic equation enabled calculations of IC_50_s indicating a relative order of toxicity of: naphthalene >xylene >toluene >benzene (Table 3). In each case abnormal development of polyps (floating and unattached see Fig. 1c) was observed at high concentrations of (1,000–10,000 μg l^−1^, Fig. 3) and total mortality was observed at higher concentrations.

The measured toxicity of the condensate treatments to coral larvae in the absence and presence of UV was estimated to be 39- and 93-fold more toxic than predicted by summing the contribution towards total toxicity of each measured component. This estimation was made by summing the combined toxicity of the individual components tested, including assumed toxicity of the other major components *m* + *o*-xylene ( = *p*-xylene) and C1-alkylnaphthalenes (=naphthalene) in the WAFs, which resulted in a predicted toxicity for 100% WAF of 0.85 toxic units (TU).

## Discussion

The intimate spatial association of coral reefs, banks and shoals in Australia’s NW shelf (Timor Sea) with offshore oil and gas exploration and production presents a number of unique development and conservation challenges. This is the first study to examine the effects of the water accommodated fraction of NW shelf gas condensate on the response of ecologically important, habitat forming corals and sponges. It also represents one of very few studies on coral-hydrocarbon ecotoxicology to incorporate important ecotoxicological principles such as the generation of concentration-response curves (to calculate IC_10_ and IC_50_ values) and measured concentrations^30^, which will allow toxicity to be compared between species, contaminant types and future studies for oil spill risk assessment purposes^25^.

Coral and sponge larvae exhibited large differences in sensitivity to condensate WAF following short term exposures. Coral larvae were much more sensitive, with the lowest concentration to inhibit metamorphosis (IC_10_) similar to concentrations measured in sub-surface water after the *Deepwater Horizon* spill^26^,^27^. Sponge larvae were ~50 times less sensitive than coral larvae, but exhibited sensitivities comparable to other tropical/sub-tropical adult species (fish, shrimp), urchin larvae and several temperate species^28^ exposed to WAF from NW shelf light crude (of similar composition to the WAF condensate tested here).

Comparing the effects of hydrocarbons on corals and sponges between studies is hindered by differences in the composition of the oils tested, the protocols used for WAF preparation, the exposure regimes and experimental durations and differences in the species and life-history stages tested. Comparisons are also challenging because most oil exposure tests with corals and sponges have reported nominal concentrations and the individual components of the oil have not been measured (Table 1). Despite these limitations, some emergent generalizations are that (i) the coral metamorphosis assay described here showed effects at similar concentrations to those previously reported for the effects of crude oil and produced formation water on coral larvae^30^,^31^, and (ii) metamorphosis of coral larvae increasingly appears to be one of the more sensitive life stages of coral to hydrocarbon exposure. For example, inhibition of larval metamorphosis in *Acropora millepora* was twice as sensitive as fertilization to crude oil WAF^31^, and most IC_50_s and LOECs reported for metamorphosis inhibition by WAF concentrations (Tables 1 and 3) are an order of magnitude lower than LC_50_s for coral larval mortality^32^. Peters and colleagues^33^ reported that adult corals can survive for 12 weeks at WAF total hydrocarbon concentrations up to 2,800 μg l^−1^, in contrast to short-term (48 h) exposures to WAFs of commercial lubricants which caused mortality of adult corals at concentrations as low as 190 μg l^−1^ THC^34^. Metamorphosis in the sponge *C. cramb*e larvae was inhibited by a PAH mixture at concentrations far lower (0.5 μg l^−1^) than TPAH concentrations reported here for corals and sponges^35^. The apparent high sensitivity of *C. crambe* could be a species-specific response or due to a range of methodological factors including differences in contaminant composition, in particular the more toxic PAH components.

The general mode of hydrocarbon (BTEX and PAH) toxicity to animals is considered to be non-specific narcosis^36^; however, the high sensitivity detected in the coral larval metamorphosis assay indicates more specific disruption of this key life history transition. The larvae of many coral species, including *A. tenuis* and *A. millepora*, will not undergo metamorphosis without an external chemical signal (cue) which is primarily derived from crustose coralline algae^37^,^38^. Components of the WAF may affect one of many critical processes from cue recognition to subsequent signal transduction or regulation of genes and biochemistry that control the metamorphosis process. The observation that some larvae underwent partial metamorphosis (changed body plan) without attachment indicates processes earlier in this life cycle transformation were affected^39^.

Exposure to UV (280–400 nm) light increased the sensitivity of the coral larvae (but not the sponge larvae) to the WAF of condensate by ~43%. UV exposure can aid in the degradation of toxic PAHs (photolysis) but can also considerably enhance PAH toxicity (photoactivation) by oxygen radical formation^29^. Previous research has indicated a 12–50,000-fold increase in toxicity to marine invertebrates from PAH photo enhancement^40^. Why the effect was less pronounced for coral larvae is unclear but could be due to a low concentration of PAHs in the tested WAF which was dominated by 2-ringed naphthalenes that exhibit far lower photoactivation than many 3- and 4-ringed PAHs^29^. BTEX compounds are not thought to be photoactivated by UV^29^. Clearly UV exposure is another important consideration which needs to be taken into account in future studies examining oil toxicity to tropical organisms in shallow, clear-water environments.

The necrotic toxicity of BTEXs and PAHs can be predicted based on their octanol/water partition coefficient, log K_ow_ (most water soluble benzene = 1.94, toluene = 2.48, *p*-xylene = 3.05 and least water soluble naphthalene =3.26)^36^,^41^. A strong inverse linear correlation was observed between the log K_ow_s of the individual contaminants tested here and their respective IC_50_s (r^2^ = 0.83, p = 0.089), indicating similar modes of action and toxicities close to those predicted for narcotic effects^28^,^41^. The sensitivity of coral larvae to condensate WAF however, was far greater than that of the major individual components of condensate WAF (IC_50_s 2,000–80,000 μg l^−1^, Table 3). This difference between measured and expected condensate toxicities was confirmed by summing the potency × concentration of each of the WAF components and calculating Σ_i_ (C_i_ / IC_50,i_) = 0.85 TU for the 100% WAF. The condensate WAF was then calculated to inhibit 50% metamorphosis at 0.026 TU, indicating an observed potency ~40-fold greater than predicted (50% inhibition should occur at 1 TU^25^). Although the narcotic effects of BTEX and PAH components are usually considered additive, non-additive effects including synergism are often observed in developmental stages of organisms^42^. It is also possible that minor components not detected or reported in the WAF analysis (e.g. trace 3-ringed PAHs or hydrogen sulphide), may have been responsible for disrupting specific developmental processes as discussed above.

The condensate used in this experiment was a partially refined fraction of components that may be found in a light crude oil blowout or spill. The composition and toxicity would be different if unrefined hydrocarbons enter the water from sub-surface blowouts or surface spills, are exposed to weathering processes or if oil spill control agents are used to disperse the hydrocarbons into the water column^43^. In this study we used 24 h exposures which may not have allowed sufficient time for maximum uptake of toxic components into the small larvae^44^. However, 24 h is likely to represent a more environmentally relevant exposure duration to the highly volatile aromatics associated with light crude than standardized 48 or 96 h chronic exposures commonly suggested for testing toxicity of early life stages^44^.

## Conclusions

Corals and sponges represent ecologically important, habitat forming tropical reef taxa and adverse effects on reproduction and recruitment via exposure to uncontrolled hydrocarbon releases would have considerable long term consequences for reef maintenance or reef recovery following disturbance. Towards more environmentally relevant testing of WAF of gas condensate from the Australian NW shelf (i.e. using tropical rather than temperate species), we have described effects on larval metamorphosis at environmentally relevant concentrations^26^,^27^, shown that toxicity is enhanced following UV exposure and demonstrated that coral larvae are more sensitive to condensate WAF toxicity than sponge larvae. Further testing on a range of relevant tropical taxa using the approach defined here would enable generation of species sensitivity distributions to more comprehensively predict and manage the risks posed by oil spills in tropical reef systems.

## Materials and Methods

### Stabilized condensate and hydrocarbons

The stabilized condensate was typical of that produced by bringing natural gas hydrocarbons from the Browse Basin of the North West Shelf, Australia to room temperature and atmospheric pressure. Stabilized condensate (also referred to as natural gasoline or distillate) of this type has a similar behaviour and composition to Type I light crude oil (Table S1). Samples were obtained in a sealed steel drum and stored at room temperature until use. The condensate had a density of 0.75 g ml^−1^ and a viscosity of ~0.65 cP. Benzene, toluene, *p*-xylene and naphthalene were purchased from Sigma-Aldrich and all had purities ≥99%.

### Water accommodated fraction preparation

Water accommodated fractions (WAF) were prepared with fresh condensate, benzene, toluene, *p*-xylene or naphthalene according to recommendations by^45^,^46^. Briefly, 800 ml 0.45 μm-filtered seawater (36 PSU, pH 8.1) was added to a solvent-rinsed I l glass aspirator bottle and mixed using a magnetic stirrer to generate a 20–25% vortex. Condensate or pure aromatics were added to the centre of the vortex at a 1:100 ratio (8 ml) and the aspirator was loosely capped and the fluids mixed for 18 h in darkness. No evidence of emulsion or bubble formation was observed after 10 min settling, so the WAF was allowed to settle for 1 h before immediate water sampling for analysis and application in the toxicity tests. Eight dilutions of the 100% WAF were prepared using the same 0.45 μm-filtered seawater to mimic dilution in the water column^47^, rather than the variable loading technique of CROSERF which is designed to mimic uptake from surface slicks^45^. The serial dilution method was chosen to maintain consistent proportions of each component in the ten treatment levels and to enable more treatment levels to be tested for improved concentration-response modelling. The National Research Council considers both approaches valid^48^.

### Hydrocarbon analysis

Hydrocarbon analyses were performed at ChemCentre (Perth, Western Australia). BTEX analyses were based on USEPA method 8260. WAF samples were analysed directly from sealed vials using Purge and Trap (P&T) GC-MS in selective ion monitoring (SIM) mode. Condensate was diluted in dichloromethane (DCM) and analysed by P&T GC-MS in scan mode. Internal standards (chlorobenzene-d_5_, 2-fluorobenzene, and 1,4-dichlorobenzene-d_4_) were added by the P&T system immediately before analysis. A method blank and a spiked control (de-ionised water with a known amount of BTEX added) were run with each sample batch. Non-quantitative whole oil analysis was performed using GC-MS directly injected with 1 μL undiluted condensate. A pre-characterised crude oil was used as a reference for hydrocarbon identification.

PAHs were analysed based on USEPA method 8270. WAF samples (500 ml) were extracted three times with DCM, the combined extracts (80 ml) were dried with sodium sulphate and 8 ml aliquots were concentrated to 1 ml under nitrogen gas. The concentrated WAF extracts and condensate (diluted in DCM) were analysed using GC-MS in scan mode. Surrogate standards (2-fluorobiphenyl, nitrobenzene-d_5_, and *p*-terphenyl-d_14_) were added to the WAF samples before extraction, and internal standards (naphthalene-d_8_, acenaphthene-d_10_, phenanthrene-d_10_, chrysene-d_12_, and perylene-d_12_) were added to the extracts and diluted condensate before analysis. A method blank and a spiked control (de-ionised water with a known amount of acenaphthene and pyrene) were run with each sample batch.

### Coral and sponge collection and larval culture

Colonies of the common Indo-Pacific broadcast spawning coral *Acropora tenuis* (Dana, 1846) > 20 cm were collected from 3–5 m depth in November 2014 from Trunk Reef, on the central mid-shelf Great Barrier Reef (GBR, 18.329°S, 146.846°E). Gravid colonies were transported to the National Sea Simulator (SeaSim) at AIMS in Townsville and placed in flow-through tanks at ~28 °C until spawning. Gametes were collected from 6 parental colonies, fertilized and the symbiont-free larvae were cultured at less than 500 larvae l^−1^ in flow through tanks as previously described^31^. *A. millepora* larvae reach maximum competency for settlement after six days and remain competent for over a month (reviewed in Jones *et al.* 2015^49^). Consequently, to ensure consistent settlement and metamorphosis, 7 to 13-day old larvae were used in separate exposure experiments. *Rhopaloeides odorabile* (Thompson, Murphy, Bergquist & Evans, 1987) is a common, viviparous, gonochoristic, dictyoceratid sponge (Demospongiiae), that broods parenchymellae larvae that are released annually over a 5–6 week period in the summer months on the GBR January^50^,^51^. Ten whole female sponges were collected from Davies Reef, central GBR Australia (18.843°S, 147.627°E) on the 11^th^ January 2015 and transported to the SeaSim at AIMS. Sponges were maintained in flow-through aquaria which allowed the controlled collection of larvae over several hours during their afternoon release. The brooded larvae were collected using larval traps, following established methods^50^. Since *R. odorabile* larvae reach maximum competency to settle after 1- 2 d^52^, experimental exposure was conducted with 24 h old larvae.

### Settlement assays

Coral and sponge larvae were each exposed to stabilized condensate WAFs, while coral larvae were additionally exposed to WAFs of the pure aromatics. Static exposures were conducted in 7 ml glass vials containing 8–10 coral larvae or 20–30 sponge larvae made up to 6.5 ml with the 10 WAF dilutions (100%, 50%, 25%, 12.5%, 6.25%, 3.125%, 1.6%, 0.8%, 0.4%, 0% WAF) with six replicate vials used for each WAF concentration. Vials were sealed with caps and a ~0.5 ml headspace enabled oxygen exchange (>7.5 mg l^−1^ over 24 h exposures) and discouraged settlement of larvae, which was often observed in trials with no head-space.

Vials were transferred to an incubator/shaker and incubated under a light level of 40 μmol quanta m^−2^ s^−1^ over a 12:12 h L:D cycle, to maintain gentle water movement. Vials were removed after a 24 h exposure and the larvae and WAF from individual vials transferred directly into individual 6-well cell culture plates (12 ml, Nunc, NY, USA). Prior to sponge settlement assays, culture plates were immersed in flow through aquaria for 48 h to develop a microbial biofilm required for successful settlement^53^. To test the potential UV-activation of PAH toxicity a duplicate series of condensate WAF exposures of coral and sponge larvae were exposed to full sunlight by immersing in a 28 °C water bath for 2 h (UVA + UVB ranged between 4.5 and 6.8 mW cm^−2^ over this period using a Solartech UV Radiometer).

For coral larvae, settlement and metamorphosis was initiated by the addition of a slightly sub-optimal (to maximise the sensitivity of the assay) concentration (10 μl) of crustose coralline algae extract^54^ prepared using 4 g of the crustose coralline algae *Porolithon onkodes*^37^. Metamorphosis was assessed after 18 h and larvae scored as normal if they had changed from either the free swimming or casually attached pear-shaped forms to squat, firmly attached, disc-shaped structures with pronounced flattening of the oral–aboral axis and with septal mesenteries radiating from the central mouth region^37^. Metamorphosis of sponges was assessed after 48 h and scored as normal if larvae had firmly attached to the surface and undergone flattening of the body to form a disc-like morphology with the centre showing the remnants of the posterior larval pole^50^.

### Data analysis

Inhibition of metamorphosis (inhibition % relative to 0% WAF control) was calculated from treatment data as Inhibition (%) = 100 × [(% metamorphosis_control_ – %metamorphosis_treatment_)/% metamorphosis_control_]. As the larval toxicity is most likely to be due to the BTEX and PAHs^25^,^44^, the concentration-response curves were generated with reference to total petroleum aromatic hydrocarbons (TPAH), which in the context of this study, is defined as the sum of total BTEX and PAHs (ΣBTEX + ΣPAHs). The concentration of TPAH that inhibited 10% and 50% of metamorphosis (IC_10_ and IC_50_) was calculated from concentration-response curves (four-parameter logistic models) fitted to the % inhibition and total aromatics data of each treatment using the program GraphPad Prism (v6, San Diego, USA). The model was constrained between 0 and 100% and all curves were tested for normality of the residuals and a replicate test was applied to assess the goodness of fit. The probability that IC_50_ values generated by the logistic curves were statistically different was tested by applying the F test in Graph Pad Prism v6. IC_50_s were considered different when p < 0.05. One-way analysis of variance (ANOVA) was performed to identify treatments which caused significant (p < 0.05) inhibition of metamorphosis in comparison with control treatments (NCSS v9, Utah, USA).

The toxicity of aromatic hydrocarbons are considered additive^44^ and by combining the concentrations and toxicities of individual compounds, the overall toxicity of a mixture can be predicted. The experimentally derived toxicity and measured concentrations of four main components of the WAF (benzene, toluene, *p-*xylene and naphthalene) were combined to determine the contribution of these components to the measured toxicity of condensate to coral larvae. This comparison was made by summing the ratios of each component’s concentration by its toxicity TU = Σ_i_ (C_i_/IC_50,i_), where TU = toxic unit, C_i_ = concentration of each component in the 100% WAF = C_i_ and the IC_50_ was calculated from concentration response curves. If the total TU of the mixture is 1 or greater an effect on metamorphosis of 50% or more would be expected^25^.

## Additional Information

**How to cite this article**: Negri, A. P. *et al.* Acute ecotoxicology of natural oil and gas condensate to coral reef larvae. *Sci. Rep.* **6**, 21153; doi: 10.1038/srep21153 (2016).

## Supplementary Material

Supplementary Information

## Figures and Tables

**Table 1:** Summary of laboratory-based studies testing hydrocarbon toxicity to reproduction and early life stages of corals and a sponge.

Species	Hydrocarbon	Endpoint	WAF Chemistry	Effect concentration (WAF% or μg l^−1^)	Ref.
Coral					
* Stylophora pistillata*	Crude oil	Mortality, metamorphosis	No	LOEC Survival: 1% LOEC Meta: 1%	23
* Pocillopora damicornis*	Unleaded gasoline:engine oil (50:1) and benzene	Mortality, metamorphosis	No	Not consistently observed	55
* Acropora tenuis*	Fuel oil 467	Fertilization	THC	LOEC Fert: 2	56
* Acropora millepora*	Heavy crude oil	Fertilization, metamorphosis	THC	LOEC Fert: 165 LOEC Meta: 82	31
* A. millepora*	Produced formation water	Fertilization, metamorphosis	THC	LOEC Fert: 72 LOEC Meta: 36	31
* S. pistillata*	crude oil	Metamorphosis, morphology	No	LOEC Meta: 0.1%, LOEC Deform.: 1% WAF	57
* A. tenuis*	Fuel oil 467	Larval survival	THC	LC_50_ 3800	32
* Goniastrea aspera*				LOEC 6800	
* Platygyra sinensis*				No observed effect	
* A. microphthalma*	Marine lubricants	Fertilization	THC	LOEC: 200	34
* P.damicornis*	Natural gas condensate	Larval mortality, Metamorphosis, Juvenile growth	No	Mortality: > 100% Growth: > 100%	58
* Pocillopora verrucosa*				Mort: > 100%, LOEC Meta: 100%, Growth: 100%	
* Seriatopora hystrix*				Mort.: 100% LOEC Meta: 50% Growth: 100%	
* S.guttatus*				Mort.: 100%, LOEC Meta: 50%, Growth: 10%	
* S. pistillata*				Mort.:0 > 100%, LOEC Meta: 100%, Growth: 10%	
* P. damicornis*	Natural gas condensate	Larval abortion (early release)	No	Abortion: 50%	59
* Montastraea faveolata*	Light crude oil	Metamorphosis, swimming, & mortality	THC	LOEC Mort: 650 LOEC Meta: 650	30
* Porites astreoides*		Larval mortality		LOEC Survival: 620 LOEC Meta: 620	
* Agaricia humilis*	Seawater from a light crude spill and Lab WAF	Larval mortality, metamorphosis	THC	Mortality: 550 Meta: 145	60
* Orbicella faveolata*				Mortality: 135	
				Meta: 135	
Sponge					
* Crambe crambe*	PAH mix	Larval metamorphosis	No	LOEC Meta: 0.5	35

Where LOEC = lowest observed effect concentration, WAF = water accommodated fraction, Met. = metamorphosis, Fert. = fertilization, Deform. = deformation, Mort = mortality. LC_50_ is the lethal concentration for 50% mortality. THC = total hydrocarbons.

**Table 2:** Concentrations of BTEX and PAHs detected in the water accommodated fractions (WAF) of North West Shelf Condensate and of individual aromatic hydrocarbons used in the current study.

Analyte	100% Condensate WAF (coral)			100% Condensate WAF (sponge)			100% Benzene WAF (coral)		100% Toluene WAF (coral)		100% *p*-Xylene WAF (coral)		100% Naphthalene WAF (coral)	
	Initial	Final −UV	Final + UV	Initial	Final −UV	Final + UV	Initial	Final	Initial	Final	Initial	Final	Initial	Final
benzene	4,500	4,000	1,100	6,967	6,200	6,100	630,000	465,608						
toluene	6,100	3,900	1,100	17,000	9,300	8,100			140,000	100,000				
ethylbenzene	140	110	29	360	310	290								
*m*,*p*-xylene	1,300	830	260	2,867	1,200	2,000					36,000	41,000		
*o*-xylene	680	460	130	650	590	820								
naphthalene	86	58	72	105	104	98							15,000	NA
C1-naphthalenes	67	49	60	90	76	79								
C2-naphthalenes				29	24	24								
fluorene	1	1												
phenanthrene	1													
Total BTEX	12,720	9,300	2,619	27,843	17,600	17,310	630,000	465,608	140,000	100,000	36,000	41,000		
Total PAH	157	107	132	224	203	201							15,000	NA
*TPAH*	*12,877*	*9,407*	*2,751*	*28,067*	*17,803*	*17,511*	*630,000*	*465,608*	*140,000*	*100,000*	*36,000*	*41,000*	*15,000*	*NA*
Mean total aromatics (24 h)		11,142	7,813		22,935	22,789		547,804		120,000		38,500		15,000

All concentrations are in μg l^−1^. NA = not analysed.

**Table 3:** Concentrations of TPAH (ΣBTEX + ΣPAHs) that inhibit 50% (IC_50_) and 10% (IC_10_) metamorphosis of coral and sponge larvae (*95% CV intervals*) calculated from dose-response curves presented in Figs 1 and 3.

	Coral condensate		Sponge condensate		Coral			
	−UV	+UV	−UV	+UV	Benzene	Toluene	Xylene	Naphthalene
IC_50_	339^a^	132^b^	~16,000	~13,000	80,351	15,559	3,939	2,077
	*(271–424)*	*(118–148)*	*(NA)*	*(NA)*	*(70,661–91,369)*	*(13,054–18,543)*	*(3423–4532)*	*(1694–2548)*
IC_10_	103	64	NA	NA	31,087	8,282	2,160	1,285
	*(63–168)*	*(49–84)*	*(NA)*	*(NA)*	*(23,508–41,109)*	*(5,416–12,666)*	*(1,576–2,960)*	*(797–2,070)*
R^2^	0.871	0.943	0.833	0.867	0.920	0.831	0.891	0.733
NOEC	180	63	11,000	11,000	34,000	15,000	4,800	1,900
LOEC	350	130	23,000	23,000	69,000	30,000	9,600	3,800
ANOVA F_9_	34.6	52.3	26.9	26.9	61.4	33.3	46.4	27.3
Meta. (%) in controls	79	75	82	90	84	70	76	67
	*(±4)*	*(±5)*	*(±6)*	*(±5)*	*(±4)*	*(±4)*	*(±4)*	*(±4)*

Different superscripted letters indicate statistically different IC_50_ values for condensate toxicity to coral larvae (F_1,120_ = 29.2, p < 0.0001). Estimates of the IC_50_s for sponge larval metamorphosis were made from limited data points on the slopes of the curves and IC_10_s were not calculated for that reason (NA). No significant observed effect concentrations (NOEC) and lowest significant observed effect concentrations (LOEC) are reported (one-way ANOVA, p < 0.05). Meta. = mean metamorphosis of control larvae in uncontaminated conditions ( ± SE).
