# Supplementary material for: Acute ecotoxicology of natural oil and gas condensate to coral reef larvae
Source: Sci Rep. 2016 Feb 19;6:21153. doi: 10.1038/srep21153 (PMC4759697; doi:10.1038/srep21153)
Supplement: Supplementary Information [file srep21153-s1.pdf]

Supplementary Information

Acute ecotoxicology of natural oil and gas condensate to coral reef larvae

Andrew P. Negri, Diane L. Brinkman, Florita Flores, Emmanuelle S. Botté, Ross J. Jones, Nicole S. Webster

Table S1. Concentrations of BTEXs, PAHs and alkyl PAHs in stabilised condensate determined using GC-MS. Concentrations are expressed as mg kg^{-1} unless otherwise indicated.

Analyte	mg kg^{-1}
benzene	2500
toluene	12000
ethylbenzene	1300
m,*p*-xylene	11000
o-xylene	1400
naphthalene	630
C1-alkylnaphthalenes	1960
C2-alkylnaphthalenes	2100
C3-alkylnaphthalenes	1200
C4-alkylnaphthalenes	<500
acenaphthylene	<50
acenaphthene	<50
fluorene	60
dibenzothiophene (DBT)	<50
C1-alkylDBTs	<500
C2-alkylDBTs	<500
C3-alkylDBTs	<500
phenanthrene	50
anthracene	<50
C1-alkylphenanthrenes	<500
C2-alkylphenanthrenes	<500
C3-alkylphenanthrenes	<500
fluoranthene	<50
pyrene	<50
C1-alkylpyrenes/fluoranthenes	<500
C2-alkylpyrenes/fluoranthenes	<500
C3-alkylpyrenes/fluoranthenes	<500
benz(a)anthracene	<50
chrysene	<50
C1-alkylbenz(a)anthracenes/chrysenes	<500
C2-alkylbenz(a)anthracenes/chrysenes	<500
benzo(b+k)fluoranthene	<50
benzo(a)pyrene	<50
C1-alkylbenzopyrenes	<500
C2-alkylbenzopyrenes	<500
indeno(1,2,3-cd)pyrene	<50
dibenz(a,h)anthracene	<50
benzo(ghi)]perylene	<50
Σ BTEXs (g kg^{-1})	28.2
Σ PAHs (g kg^{-1})	6.0
TPAH (Σ BTEX + Σ PAH; g kg^{-1})	34.2

Table S2. Concentrations of BTEXs, PAHs, alkyl PAHs and TRH in solvent-extracted 100% condensate WAFs used for the coral and sponge larvae experiments determined by GC-MS. Concentrations are expressed as µg l^{-1}.

Analyte	Coral	Sponge
benzene	4500	6967
toluene	6100	17000
ethylbenzene	140	360
m,p-xylene	1300	2867
o-xylene	680	650
naphthalene	87	105
C1-alkylnaphthalenes	68	90
C2-alkylnaphthalenes	<0.5	29
C3-alkylnaphthalenes	<0.5	<0.5
C4-alkylnaphthalenes	<0.5	<0.5
acenaphthylene	<0.1	<0.1
acenaphthene	<0.1	<0.1
fluorene	1	<0.1
dibenzothiophene (DBT)	<0.1	<0.1
C1-alkylDBTs	<0.5	<0.5
C2-alkylDBTs	<0.5	<0.5
C3-alkylDBTs	<0.5	<0.5
phenanthrene	1	<0.1
anthracene	<0.1	<0.1
C1-alkylphenanthrenes	<0.5	<0.5
C2-alkylphenanthrenes	<0.5	<0.5
C3-alkylphenanthrenes	<0.5	<0.5
fluoranthene	<0.1	<0.1
pyrene	<0.1	<0.1
C1-alkylpyrenes/fluoranthenes	<0.5	<0.5
C2-alkylpyrenes/fluoranthenes	<0.5	<0.5
C3-alkylpyrenes/fluoranthenes	<0.5	<0.5
benz(a)anthracene	<0.1	<0.1
chrysene	<0.1	<0.1
C1-alkylbenz(a)anthracenes/chrysenes	<0.5	<0.5
C2-alkylbenz(a)anthracenes/chrysenes	<0.5	<0.5
benzo(b+k)fluoranthene	<0.1	<0.1
benzo(a)pyrene	<0.1	<0.1
C1-alkylbenzopyrenes	<0.5	<0.5
C2-alkylbenzopyrenes	<0.5	<0.5
indeno(1,2,3-cd)pyrene	<0.1	<0.1
dibenz(a,h)anthracene	<0.1	<0.1
benzo(ghi)]perylene	<0.1	<0.1
Σ BTEX	12720	27843
Σ PAH	157	224
TPAH (Σ BTEX + Σ PAH)	12877	28067

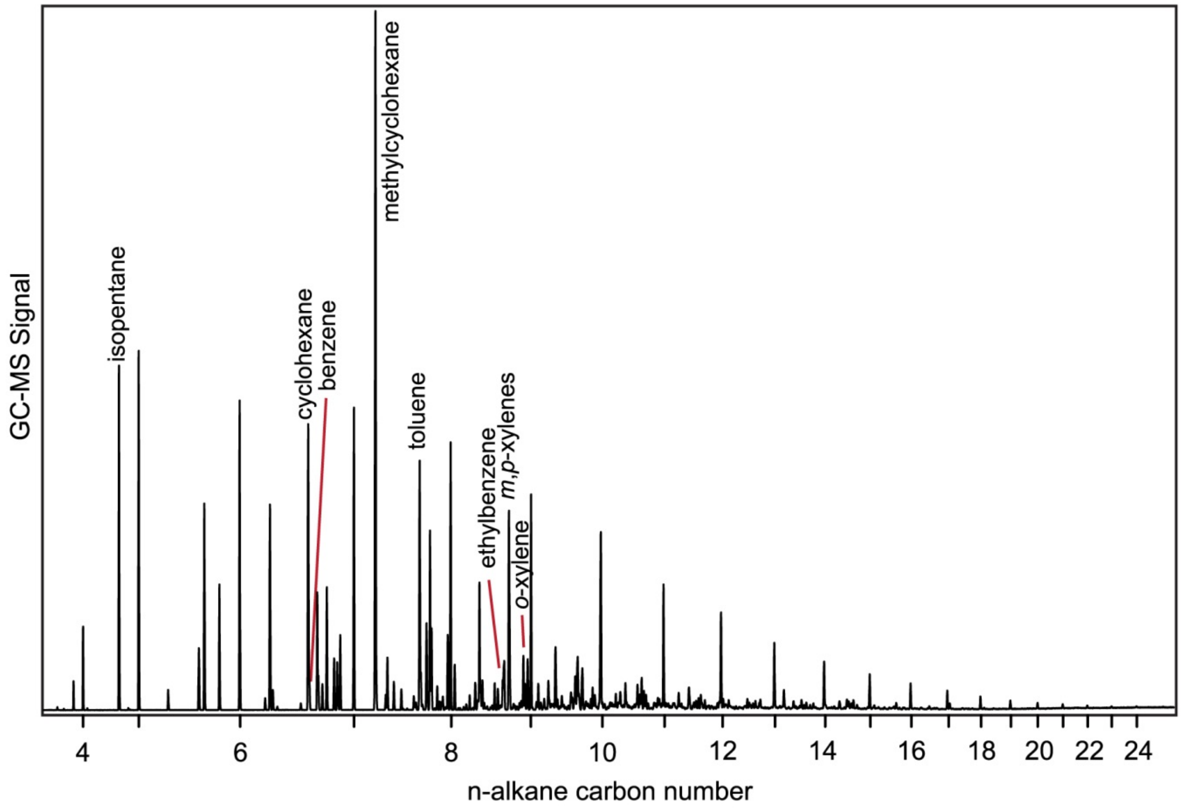

Figure S1. GC-MS chromatogram of stabilised condensate (direct injection). On the *x*-axis, retention time has been converted to n-alkane carbon number.

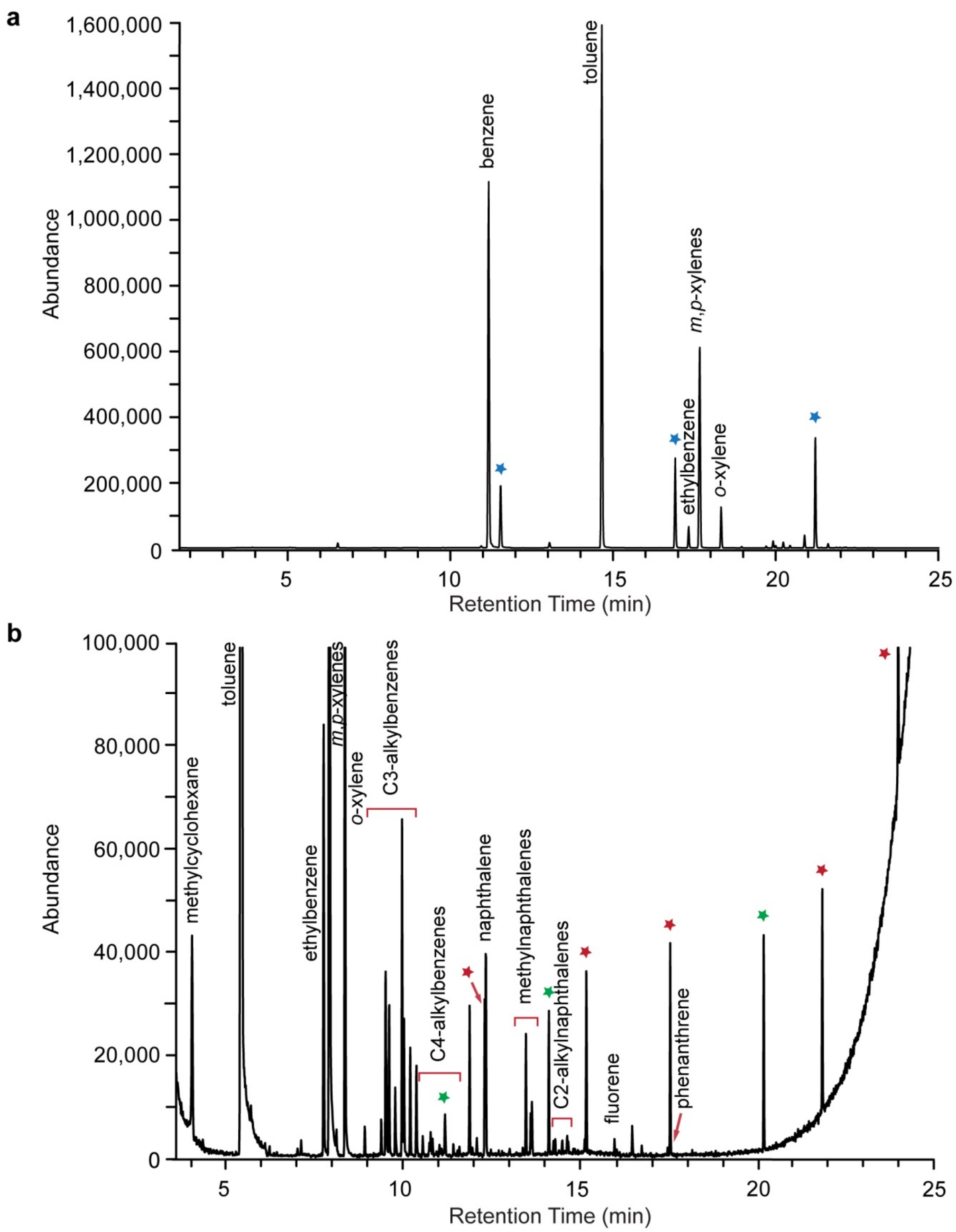

Figure S2. Chromatograms of condensate WAF used in coral exposures. a) Purge and trap GC-MS (SIM) for quantification of BTEXs; and b) flash vaporization GC-MS (full scan) for quantification of PAHs and alkyl-substituted PAHs (alkylbenzenes and methylcyclohexane are also indicated but were not quantified). Internal (★,★) and surrogate (★) standards are listed in order of elution: ★ = chlorobenzene-d_5, 2-fluorobenzene and 1,4-dichlorobenzene-d_4; ★ = naphthalene-d_8, acenaphthene-d_{10}, phenanthrene-d_{10}, chrysene-d_{12} and perylene-d_{12}; ★ = nitrobenzene-d_5, 2-fluorobiphenyl and *p*-terphenyl-d_{14}.
